# Developing a systems-focused tool for modeling lung cancer screening resource needs

**DOI:** 10.1186/s12962-024-00573-w

**Published:** 2024-09-05

**Authors:** Aparna Reddy, Fumiya Abe-Nornes, Alison Haskell, Momoka Saito, Matthew Schumacher, Advaidh Venkat, Krithika Venkatasubramanian, Kira Woodhouse, Yiran Zhang, Hooman Niktafar, Anthony Leveque, Beth Kedroske, Nithya Ramnath, Amy Cohn

**Affiliations:** 1 University of Michigan, Ann Arbor, MI USA; 2 Veterans Affairs Ann Arbor Healthcare System, Ann Arbor, MI USA; 3 Rogel Cancer Center, Michigan Medicine, Ann Arbor, MI USA

**Keywords:** Interventions, Lung cancer screening program, Resource allocations, Screening, Veterans affairs

## Abstract

**Background:**

Early detection through screening dramatically improves lung cancer survival rates, including among war Veterans, who are at heightened risk. The effectiveness of low dose computed tomography scans in lung cancer screening (LCS) prompted the Veteran’s Affairs Lung Precision Oncology Program (VA LPOP) to increase screening rates. We aimed to develop an adaptive population health tool to determine adequate resource allocation for the program, with a specific focus on primary care providers, nurse navigators, and radiologists.

**Methods:**

We developed a tool using C + + that uses inputs that represents the process of the VA LCS program in Ann Arbor, Michigan to calculate FTEs of human resource needs to screen a given population. Further, we performed a sensitivity analysis to understand how resource needs are impacted by changes in population, screening eligibility, and time allocated for the nurse navigators’ tasks.

**Results:**

Using estimates from the VA LCS Program as demonstrative inputs, we determined that the greatest number of full-time equivalents required were for radiologists, followed by nurse navigators and then primary care providers, for a target population of 75,000. An increase in the population resulted in a linear increase of resource needs, with radiologists experiencing the greatest rate of increase, followed by nurse navigators and primary care providers. These resource requirements changed with primary care providers, nurse navigators and radiologists demonstrating the greatest increase when 1–20, 20–40 and > 40% of Veterans accepted to be screened respectively. Finally, when increasing the time allocated to check eligibility by the nurse navigator from zero to three minutes, there was a linear increase in the full-time equivalents required for the nurse navigator.

**Conclusion:**

Variation of resource utilization demonstrated by our user facing tool emphasizes the importance of tailored strategies to accommodate specific population demographics and downstream work. We will continue to refine this tool by incorporating additional variability in system parameters, resource requirements following an abnormal test result, and resource distribution over time to reach steady state. While our tool is designed for a specific program in one center, it has wider applicability to other cancer screening programs.

**Supplementary Information:**

The online version contains supplementary material available at 10.1186/s12962-024-00573-w.

## Background

Lung cancer is the deadliest cancer in the US with a 5 year survival rate of less than 5% when detected in advanced, metastatic Stage 4 cancer [1]. When detected early (e.g. Stage I) through screening using low dose computed tomography (LDCT), the 5 year survival rate increases to 60–70%, highlighting the importance of early detection [1]. Across all stages, lung cancer screening (LCS) using LDCT results in a 20–24% reduction in lung cancer specific mortality; nonetheless, the rate of LCS among those eligible is only 6% [2, 3]. Low adoption of LCS using LDCT among high-risk individuals can therefore result in lower detection of early-stage lung cancer and worse survival from lung cancer.

American war Veterans are particularly at high risk for lung cancer, due to age, smoking prevalence, and other environmental exposures related to military service [4]. There are an estimated 900,000 Veterans who are at risk for lung cancer [5]. Approximately 8,000 veterans are diagnosed and treated for lung cancer at the Veteran’s Affairs (VA) each year, and about 5,000 Veterans die from this cancer each year [5, 6]. There is a significant unmet need and opportunity for a clinical intervention to improve lung cancer survival through screening and early treatment [5–7]. Even though LCS guidelines have been in place since 2013 from the US Preventive Services Task Force (USPSTF), the lack of a nationally coordinated program led to low LCS rates among Veterans. In 2018, 12,400 Veterans were screened for lung cancer in 81 facilities across 43 States, with 5 facilities performing < 10 screening CTs. There were a median number of 96 LDCT screens for 36,848 eligible Veterans by state [2, 3, 8]. Several factors can contribute towards low adoption of LCS, such as limited shared decision-making [9], lack of patient navigators, too few radiology resources [10], and patient related factors such as low awareness of the importance of screening [11] among high-risk patients [12–15]. This prompted the VA to initiate a national lung precision oncology program (LPOP), the goals of which were to increase LCS for earlier detection of lung cancers and to offer precision oncology care for those Veterans diagnosed with lung cancer.

LPOP involves an integrated network of 23 Hub Sites tasked with increasing LCS and providing precision oncology care. The VA Ann Arbor Health System (VAAAHS) LPOP program, located in Hub Site 10 had an initial goal of increasing screening of eligible Veterans from 5% to 30%. To do this, a decentralized hub and spoke model was created with 1 hub site and 5 spoke sites, each catering to a population of over 50,000 eligible Veterans.

Although integrating cancer screening programs in existing healthcare systems has great potential in reducing patient morbidity and mortality, not all efforts have been successful [14]. Multi-level barriers to successful LCS programs include, among others, inadequate resource allocation, including insufficient staffing and financing [14, 16]. Resource allocation is a major barrier to the successful implementation of screening interventions because it creates a strain on programs and healthcare workers [9]. When resources are inadequately allocated, there is an increased likelihood of bottlenecks that prevent programs from achieving their goals [17]. Therefore, targeting poor resource allocation by defining resource needs systematically and preemptively is critical.

This study focuses on one LCS program, as it was initiated at a major academic VA medical center to understand the process and identify key resources required to initiate and sustain a robust and efficient LCS program. We focus on three supply side key human resources, namely primary care providers (PCPs), nurse navigators (NNs), and radiologists, and the tasks involved in the screening process to inform our tool. We designed an adaptive population health tool to estimate human resource needs that could be used not only to optimize LCS but could also be used in the future for a broad range of screening programs in both the initial development and implementation phase.

## Materials and methods

### General overview

**Fig. 1:**
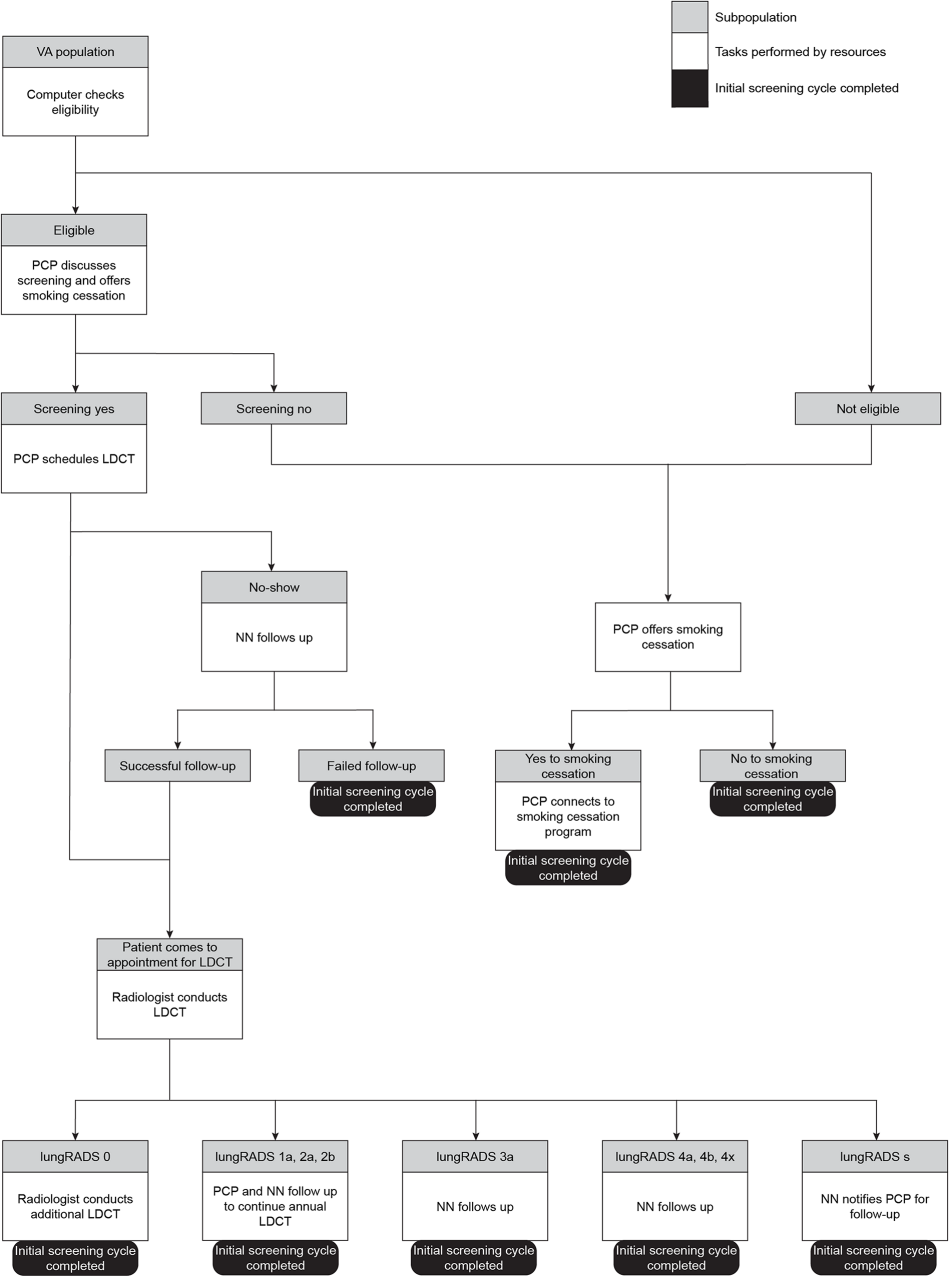
Process Map for Demonstrative Example: Lung Cancer Screening Program, VA Ann Arbor demonstrates the steps involved in lung cancer screening and key human resources involved including Nurse Navigators (NNs), primary care providers (PCPs), and radiologists, who aid in subdividing the population into subgroups by criteria until every patient in the population completes one cycle of screening in the program

The VA LCS program operates under two process models, the hybrid model and the consult model. In the hybrid model, outlined as a process map in a later section as Fig. 1, the PCP is the initial gatekeeper. The PCP is responsible for providing shared decision making with the patient, ordering the LDCT scans, and referring the patient to the nurse navigator (NN) to enter the LCS program dashboard (See Supplemental Fig. 1). The NN is responsible for following up on return of results and subsequent follow-up steps. Successful LCS results are returned as a report on the LungRADs scale of 1 (no nodules detected) to 5 (highly suspicious nodes). If the LDCT returned a score of 1 or 2, the NN enters the patient in the dashboard to be followed the subsequent year with annual LDCTs. If the LDCT revealed a suspicious finding (score of 3, 4, or 5), the PCP would inform the patient and the NN would present the patient’s LDCT at the multidisciplinary board to ascertain next steps, such as referral to the pulmonary or interventional radiology service. There is also the possibility that a patient receives a score of a LungRADs score of a 0, which indicates an incomplete scan due to the lungs being obscured, typically by either due to coexisting pulmonary disease or due to patient movement during the scan. These cases are typically rescanned after three months, although the duration is dependent on the cause for the interpretable scan. Our goal here is to examine the resourcing of such a hybrid model.

The alternative model is a consult model. In this model the PCP identifies the patient eligible for LCS and refers the patient to a NN. The NN in turn, initiates shared decision making, orders the LDCT and performs the necessary downstream steps, including return of results and referral to interdisciplinary services such as pulmonary, thoracic surgery and oncology. An in-depth examination of resources required for this model is beyond the scope of this project.

### Population inputs

The target population we chose were Veterans at high risk for lung cancer, as defined by the United States Preventive Services Task Force USPSTF 2013 (age 55–80 with a 30 pack-year smoking history who currently smoke or have quit smoking in the previous 15 years) [18].

### Human resource inputs

Our study focuses on three key human resources, Nurse Navigators (NN), Primary Care Providers (PCPs), and Radiologists. NNs are individuals who have a nursing degree or higher with experience in direct patient care and managing data. PCPs provided general medical care to the Veterans in the facility and are responsible for all aspects of screening not only for lung cancer, but also other cancers such as breast and colorectal cancer. Radiologists in the facility have special expertise in chest radiology and are required to enter LDCT results in a templated record (See Supplemental Fig. 2).

### Model assumptions

There are three assumptions which were explicitly made when developing our tool. While by principal we wish for the model to accurately reflect systems as they exist, there are simplifications and concessions which must be made and acknowledged at this point in the process.

First, we assumed that the steps of the screening program follows a linear, step wise progression where patients do not repeat any steps, such as undergoing a repeat scan during a given screening cycle when it comes to obtaining a patient’s first scan. For future models, the cyclical nature of follow-up appointments will be examined. Second, we assumed that it takes a fixed amount of time for each human resource to complete a task, with no variability from patient to patient. This amount of time is based on an average estimate of task time from collaborating persons. Third, the tool assumes an ideal scenario of 100% resource utilization.

### Development of the tool

The tool calculates the total amount of time required of each type of human resource, in Full-Time-Equivalents (FTEs) of 40 h of work per week to screen a given patient population. The steps in the process to screen a patient population consists of tasks that require time to complete by each human resource.

The tool was developed using C + + to read an input file to represent the steps and their order as described in the process map in Fig. 1. To find the resource utilization of the program, the tool follows patients through the program and tracks the resource needs per step and later aggregates them. This aggregated sum of resource needs for all steps in the program determines the total amount of a specific resource required (Formula 1).

1 $$\begin{array}{l}\:\sum\:{\text{i}}_{Step}\left(Number\:of\:Patients\:at\:Step\:i\:x\:Resource\:needs\:per\:patient\right)\\=Total\:Resource\:Requirement\end{array}$$

This formula is the fundamental logic applied in the tool. The tool uses inputs from the model depicted in Fig. 1, where the starting VA population of Veterans at risk is divided into subpopulations of eligible and non-eligible patients. Eligibility is ascertained by the PCP (using a clinical reminder tool), assisted by NN, based on criteria established by the USPSTF [18] and predicted life expectancy due to other medical comorbidities. These two sub-populations subsequently flow through a series of steps that further subdivide them. The program ends when all patients complete one cycle of screening by reaching a terminal step.

For example, a patient following a pathway through the program could be flagged as eligible by the VA patient portal, followed by shared decision making with the PCP, who then discusses LDCT based LCS and smoking cessation. If patient agrees, the PCP orders the LDCT and refers the patient to the NN to enter the dashboard. The NN follows the patient who has completed the LDCT with the results provided by the radiologist. The radiology reports follow a specific template that uses the American College of Radiology based lung imaging and reporting data system (LungRADs) score (See Supplemental Figs. 2 and 3).

### Determining inputs

Seven of the authors (AR, FA, AH, AV, KV, MS, and YZ) shadowed 2 NNs through their day, timed their tasks, and attended the multidisciplinary nodule clinic to see how high-risk nodules would be triaged for subsequent workup. We documented the time spent on each step of the program by the NNs, PCPs, and radiologists, which include eligibility assessment, screening consent, testing, and evaluation, until every patient had completed one cycle of screening within the program.

We also gathered inputs on the population, including number of patients assessed for LCS (which involves calculating tobacco pack years to assess smoking history), number of patients actively enrolled in the LCS program, number of patients appropriately screened (based on competing medical comorbidities and life expectancy), and the number of patients who received a diagnosis of potential lung cancer defined by the LungRADS scores (See Supplemental Fig. 3), and percentage of patient adherence towards annual screening. We gathered these inputs from 09/2021 to 08/2023 through the National Center for Lung Cancer Screening Metrics Dashboard (See Supplemental Fig. 1).

Our tool reads a user-defined input file with associated parameters (per-patient resource needs at each step and probability of each outcome) for the initial population. The tool then computes the number of patients passing through each step and the overall resources needed for this work.

We have compiled these inputs in Table 1. Each task is completed by a single human resource in an average amount of time, which we calculated by conducting time studies and shadowing. Task ID’s are arbitrarily assigned to each task on the process map for identification, and connections between tasks indicating sequential steps are created through “Next Task IDs”. The percentage outcome and resource needs in minutes for each Task ID are used as inputs to calculate total resource needs by the model. These percentages were calculated from the information provided by the National center for Lung Cancer Screening Metrics Dashboard.

**Table 1:** Input file for demonstrative example: VA lung cancer screening program

Initial VA Population: 75,000					
ID	Task	Resources *Minutes per patient*			Next Task ID: Percentage Outcome
		NN	PCP	Radiologist	
1	Computer checks eligibility of VA population	0	0	0	2: 30% Eligible 3: 70% Ineligible
2	PCP discusses screening and offers smoking cessation	0	3	0	3: 40% Screening no 5: 60% Screening yes
3	NN offers smoking cessation	1	0	0	4: 60% Yes smoking cessation 13: 40% No smoking cessation
4	PCP connects to smoking cessation program	0	1	0	13: 100% Initial screening cycle completed
5	PCP schedules LDCT	0	2	0	6: 10% No-show 7: 90% Patient comes for LDCT
6	NN follows up	5	0	0	7: 75% Successful follow-up 13: 25% Failed follow-up
7	Radiologist conducts LDCT	0	0	10	8: 0.5% lungRADS0 9: 49.6% lungRADS 1a, 2a, 2b 10: 41.8% lungRADS 3a 11 : 3.4% lungRADS 4a, 4b, 4x 12: 4.7% lungRADS s
8	lungRADSO: Radiologist conducts additional LDCT	0	0	10	13: 100% Initial screening cycle completed
9	lungRADS 1a, 2a, 2b: PCP and NN follow up to continue annual LDCT	10	0	0	13: 100% Initial screening cycle completed
10	lungRADS 3a: NN follows up	10	0	0	13: 100% Initial screening cycle completed
11	lungRADS 4a, 4b, 4x: NN follows up	10	0	0	13: 100% Initial screening cycle completed
12	lungRADS s: NN notifies PCP for follow up	10	0	0	13: 100% Initial screening cycle completed
13	Patients complete one cycle of screening in the program	0	0	0	N/A

Data inputs for our tool: For each task displayed in Fig. 1, the resource requirements and subsequent tasks with associated distributions are shown

## Results

**Fig. 2:**
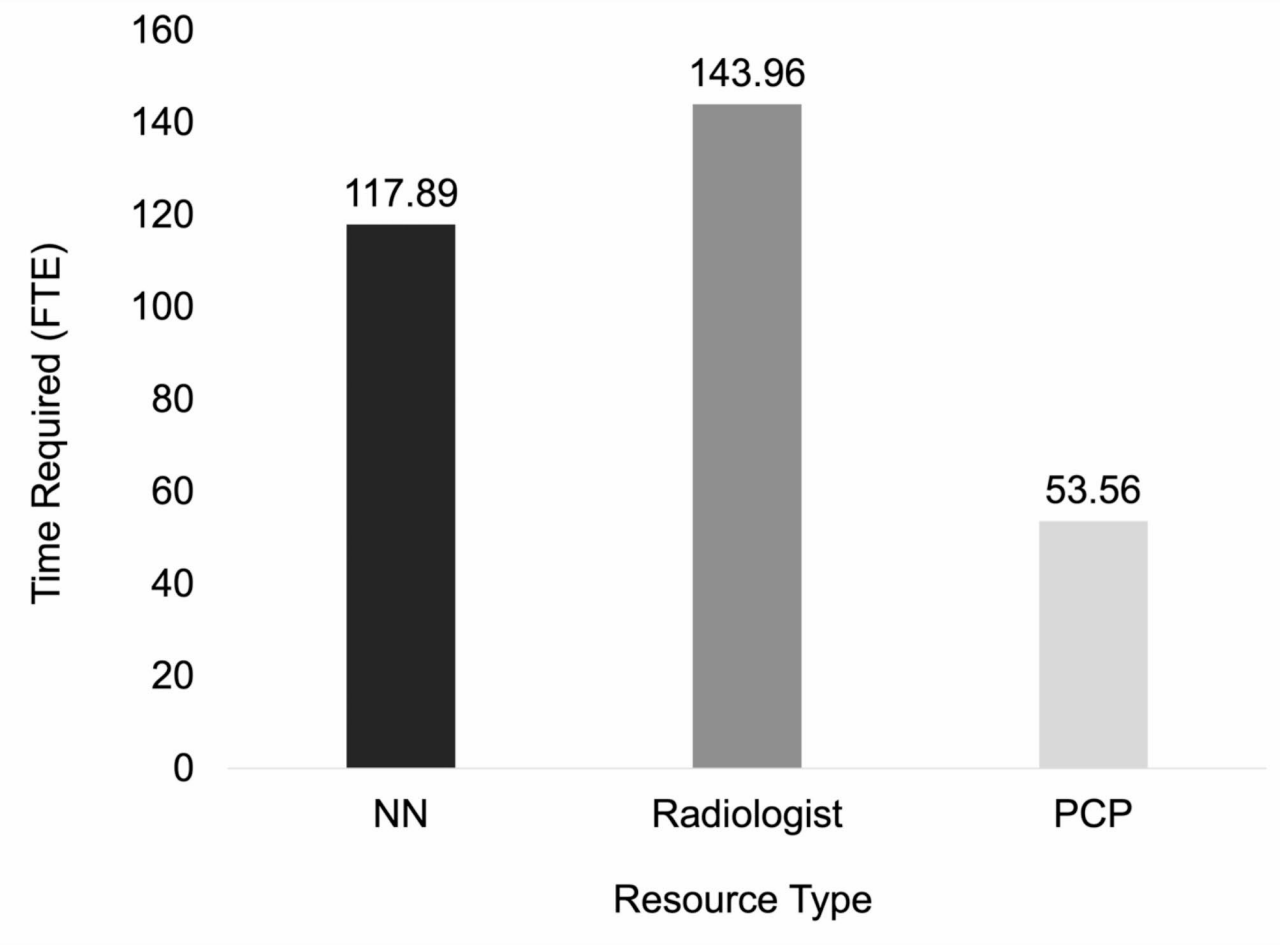
Resource Requirements for NN, Radiologist and PCP in FTEs. The number of FTEs required for each resource type based on the inputs used in Table 1

First, using the inputs from Table 1, we calculated the total time measured in units FTEs, i.e., number of people working a 40-hour week, across three resource types (PCP, NN, Radiologist). We found that the radiologist required the most FTEs of 143.96 FTEs, followed by the NN with 117.89 FTEs, and PCP requiring 53.56 FTEs (Fig. 2).

**Fig. 3:**
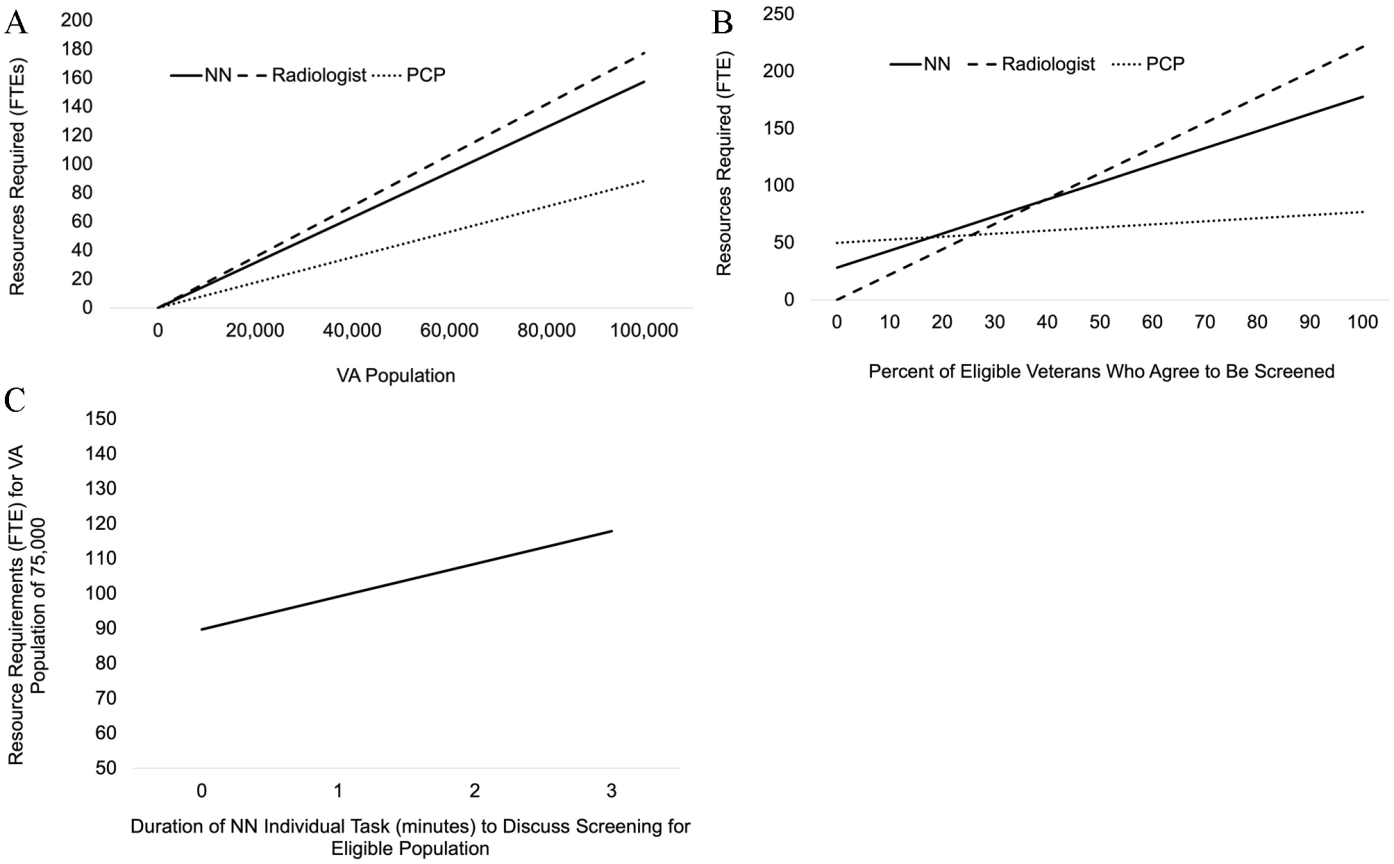
**a:** Resource Requirements for NN, Radiologist and PCP with an Increasing VA Population. **b:** Resource Requirements for NN, Radiologist and PCP with Varying Percent of Eligible Veterans. **c:** Resource Requirements for NN with Varying Task Duration to Discuss Screening. Sensitivity analysis following varying initial eligibility and task duration: **a** depicts the impact on Full Time Equivalents (FTEs) for each resource type when the initial population increases, holding all other parameters from Table 1 constant. **b** depicts the impact on Full time Equivalents (FTEs) when the percentage of eligible Veterans who agreed to be screened changes, holding the population constant at 75,000. **c** depicts the impact on Full Time Equivalents (FTEs) when the duration of the task to discuss screening changes with a fixed population of 75,000 and holding all other parameters from Table 1

Second, we considered the impact of variability across different parameters, and performed a sensitivity analysis. Holding all other inputs consistent with Table 1, we determined the impact of FTEs when the initial population was expanded (Fig. 3a), when the percentage of eligible Veterans who agreed to be screened changed (Fig. 3b), and when the duration of time allotted for the PCP/NN performing shared decision-making regarding screening changed (Fig. 3c).

As the population size increased from 75,000, the FTEs required by each resource type increased linearly, with the radiologist experiencing the greatest rate of increase in FTEs, followed by NN and PCP (Fig. 3a). Similarly, as the percentage of eligible Veterans who accept to be screened increased, the FTEs required for each resource type also increased linearly, with radiology experiencing the greatest rate of increase in FTEs (Fig. 3b). When the percent of eligible Veterans who accept to be screened is between 0 and 20%, PCPs are the most required resource. However, when the percent of eligible Veterans who accept to be screened is between 20 and 40%, NNs are the most required resource, and when the percent of eligible Veterans who accept to be screened is between 40 and 100%, radiologists are the most required resource (Fig. 3b). Finally, when increasing the duration of the task to check eligibility by the NN from the zero to three minutes, the NN experienced a linear increase in the FTEs required (Fig. 3c).

## Discussion

As LCS becomes more widespread and with a further relaxation of eligibility criteria, there is going to be a significant increase in the numbers of individuals who will be screened [19]. There is a paucity of data on both the quantification and solutions that will become necessary to equitably distribute resources required for implementation of such programs. We provide a tool that focusses on human resources needs, of PCPs, NNs and radiologists, needed at the entry point of the program.

The results from running our tool using the inputs from a newly implemented LCS program suggest that as the target patient population increases, the rate of utilization of radiologist followed by the NN increases to a greater extent respectively than the PCP. This suggests that while resource allocation will change with expanding the program, all resources may not increase at the same rate, and some may require more investment than others.

The differing rates of resource use with various eligibility denominators suggest that human resource utilization in one population with a certain eligibility rate cannot be applied to another population with a different eligibility rate, as the impact of resource use changes with variability in population demographics. This highlights the need for a critical evaluation of a population rather than a one-size-fits-all approach to better inform resource allocation.

In addition to various population demographics, the variability in different amounts of time to complete a task by one NN, or among different NNs, impacts the overall resource use. For example, variability in time to complete the task of discussing screening with the eligible population can affect resource utilization: increasing task time from 1 to 3 min, linearly increases the overall use of the NN resource in the program. Therefore, it is important to consider the amount of time taken per resource in a program to better inform resource allocation.

There are limitations of our study. First, our tool has only considered the supply side of human resources involved in the most proximal portion of a LCS program. We have not considered the human resources required, following a diagnosis of a suspicious lung nodule. The steps involved include presentation of such cases at a lung nodule multidisciplinary board attended by pulmonologists, radiologists, thoracic surgeons, medical and radiation oncologists. If a nodule requires follow-up, the NN needs to put that individual back into the database to follow through next steps. If a nodule requires further work up and need for surgery or radiation, then the patient exits the screening program, but there is still a requirement to “close the loop” by the navigator and the cancer registrar to document stage and therapy for the initial cancer and to follow the patient until death or survival at 5- years.

Second, we have not considered the administrative assistants under human resources, the ones who call the patients to make the appointments or the administrator of the program who assures that all metrics from screening are being met.

Third, we have not considered the non-human resources involved such as the CT scanner itself and how use of the scanners for screening might burden a system that is being used to deliver care for other diseases or established cancers. Alongside this, we have not considered resources needed for additional workup such as a PET scan or a bronchoscopy, as well as resources for treatment such video assisted robots for surgery and anesthesia requirements.

Regarding our methods itself, one of the limitations is that the fixed probabilities of dividing subpopulations does not allow for changes in patient-related factors over time, such as changes in tobacco smoking patterns or varying Lung-RADS scores in each patient over time – this may change the time for the NN tasks.

Another limitation of the current tool it is that it assumes an ideal scenario of 100% resource efficiency and only considers average resource utilizations and population distributions, rather than recognizing their inherent variability. This may significantly impact resource use overtime. Furthermore, while determining resource needs for a new intervention is important, it can be challenging to determine inputs for a tool that may lack prior historical data. We will continue to iterate our tool by building in confidence intervals and sensitivity to account for the confidence of the input parameters and resulting outputs.

More broadly, our model does not address temporal aspects of implementation. For example, a program such as this would not seek to screen the entire population in a single day, week, or even month. Rather, it would be designed to work through the backlog of existing unscreened individuals and then reach a steady state where newly eligible individuals would be screened on a consistent basis. This is another area of the model that we seek to improve, by incorporating additional inputs of annual changes in the population.

Furthermore, healthcare screening interventions are influenced by demand, supply, and access that directly influence screening prevalence. Future studies considering how factors such as patient education and desire to be screened, along with accessibility of screening services influence demand for screening, and therefore resource needs, can provide further insight to determine resources for a program and how it may change based on program outreach efforts.

Taken together, we have developed a provider facing modifiable tool using inputs from one LCS program where we illustrate the need for a tailored approach to resource allocation. With further refinement, this tool can be utilized for efficient planning and implementation of new or existing interventions in different healthcare settings as new preventative strategies and screening guidelines are recommended. Furthermore, this tool can be applied in resource-constrained settings to determine how to best allocate resources to maximize the impact of the intervention.

## Conclusion

Through this study, a tool was developed to improve informed decision-making for resource allocation in a lung cancer screening program. Our tool identified variability in resource utilization impacted by provider-related factors. This tool can be applied to other intervention programs to improve their program success by considering population demographics, staffing, and resource availability. In the future, the model will be enhanced to incorporate variability and timing, leading to further refinement and accuracy of the estimated resource needs.

## Electronic supplementary material

Below is the link to the electronic supplementary material.

**Supplementary Material 1**: **Supplemental Fig. 1 |** Lung Cancer Screening Program Metrics Dashboard: The dashboard provides data on the number of patients assessed for tobacco pack years (criteria for screening eligibility), number of patients actively enrolled in lung cancer screening (LCS), number of patients who have completed Low Dose Computerized Tomography Scan (LDCT), and patient adherence rate to completed screening.

**Supplementary Material 2**: **Supplemental Fig. 2** | Radiology Reporting Template: This template is used by radiologists in the lung cancer screening program to report findings of a low-dose computerized tomography scan that uses the American College of Radiology based lung imaging and reporting data system (LungRADs) score. A web simulator can be accessed here: https://assistweb.acr.org/Lung%20RADS?_gl=1*ijir2v*_ga*MTE4NjExMTYyMi4xNzEzNjQ3MjE3*_ga_K9XZBF7MXP*MTcxMzY0NzIxNi4xLjEuMTcxMzY0NzIxNi4wLjAuMA.

**Supplementary Material 3**: **Supplemental Fig. 3** | LungRADs Score Descriptions: This file describes each LungRADs score (0, 1, 2, 3, 4a, 4b, 4x, Su and S) with corresponding clinical findings and case management.

## Data Availability

Data sharing is not applicable to this article as no datasets were generated or analyzed during the current study.

## Abbreviations

FTE: Full-Time-Equivalent
LCS: Lung cancer screening
LPOP: Lung Precision Oncology Program
NN: Nurse Navigator
PCP: Primary Care Provider
VA: Veterans Affairs
VAAAHS: Veterans Affairs Ann Arbor Healthcare System
USPTFS: United States Preventive Task Force Services
