# Supplementary figures and images for: Developing a systems-focused tool for modeling lung cancer screening resource needs
Source: Cost Eff Resour Alloc. 2024 Sep 5;22:63. doi: 10.1186/s12962-024-00573-w (PMC11378520; doi:10.1186/s12962-024-00573-w)

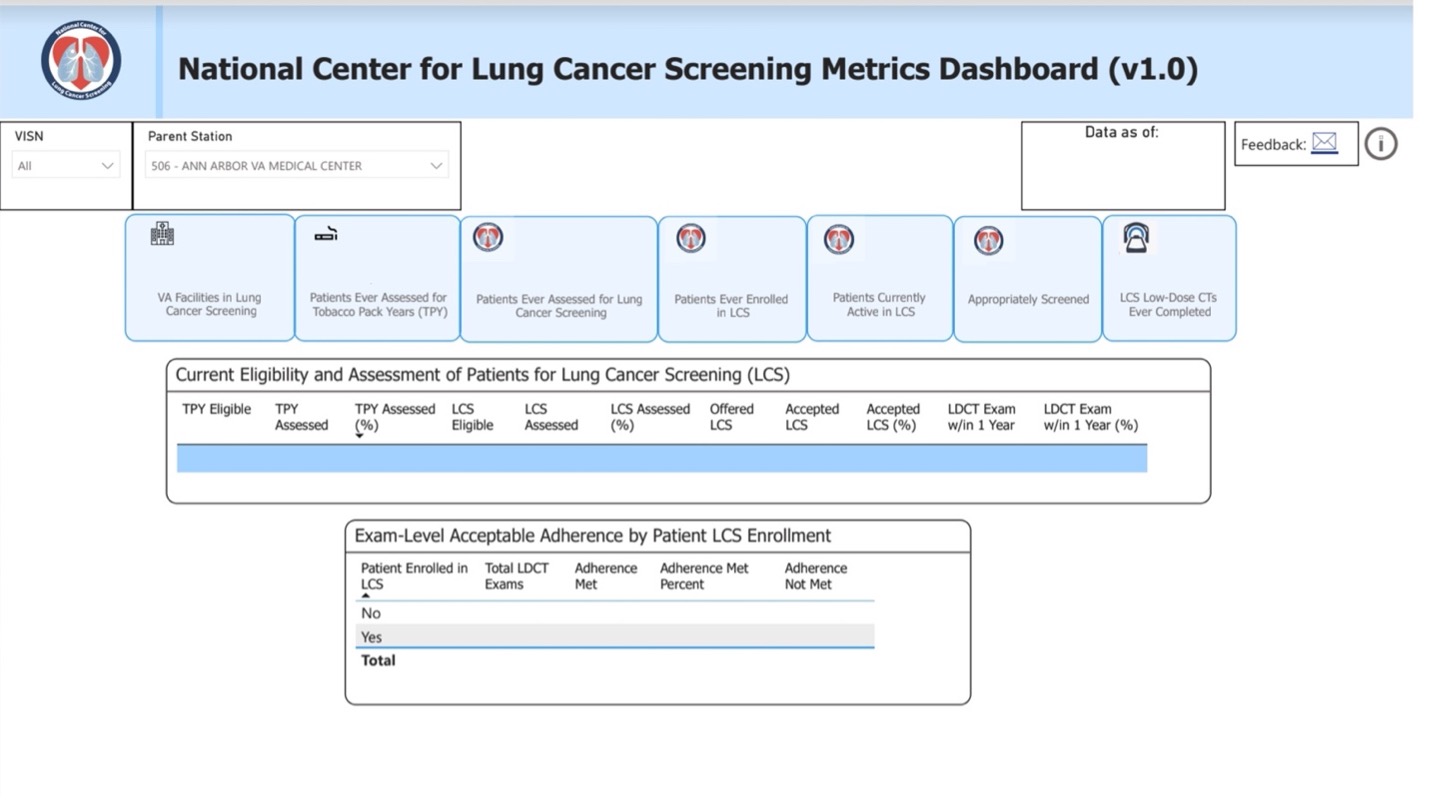

Supplement: Supplementary file 1 — Supplementary Material 1: Supplemental Fig. 1 | Lung Cancer Screening Program Metrics Dashboard: The dashboard provides data on the number of patients assessed for tobacco pack years (criteria for screening eligibility), number of patients actively enrolled in lung cancer screening (LCS), number of patients who have completed Low Dose Computerized Tomography Scan (LDCT), and patient adherence rate to completed screening. [file 12962_2024_573_MOESM1_ESM.jpg]

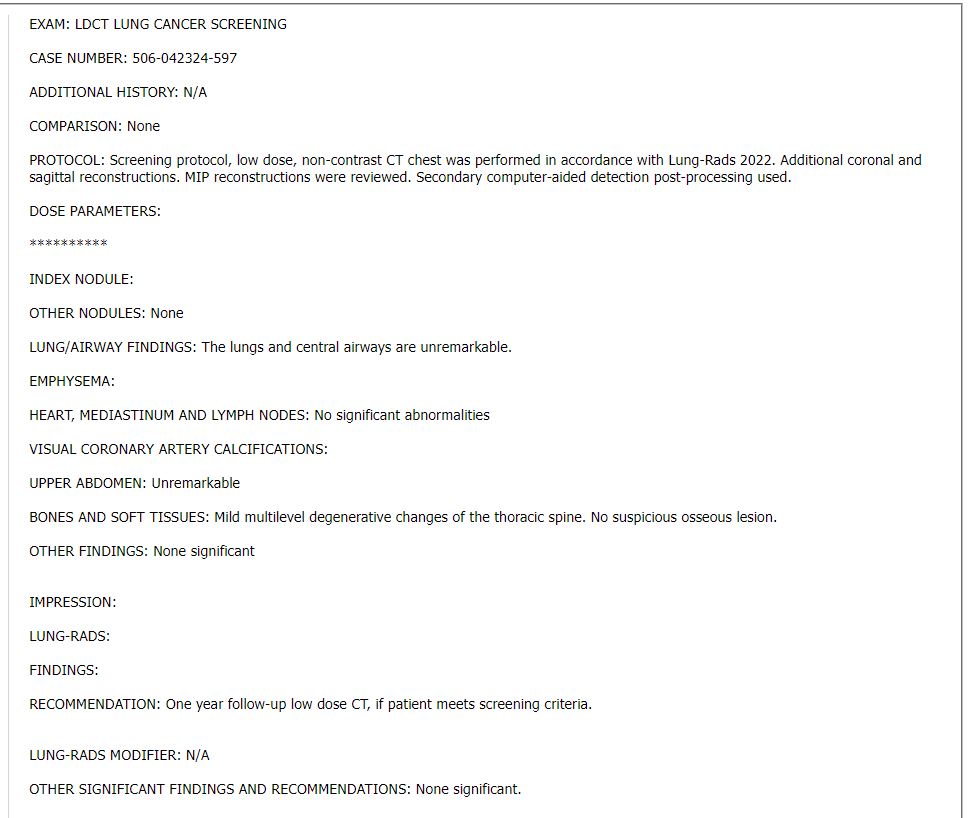

Supplement: Supplementary file 2 — Supplementary Material 2: Supplemental Fig. 2 | Radiology Reporting Template: This template is used by radiologists in the lung cancer screening program to report findings of a low-dose computerized tomography scan that uses the American College of Radiology based lung imaging and reporting data system (LungRADs) score. A web simulator can be accessed here: https://assistweb.acr.org/Lung%20RADS?_gl=1*ijir2v*_ga*MTE4NjExMTYyMi4xNzEzNjQ3MjE3*_ga_K9XZBF7MXP*MTcxMzY0NzIxNi4xLjEuMTcxMzY0NzIxNi4wLjAuMA. [file 12962_2024_573_MOESM2_ESM.jpg]
