# Supplementary material for: Developing a systems-focused tool for modeling lung cancer screening resource needs
Source: Cost Eff Resour Alloc. 2024 Sep 5;22:63. doi: 10.1186/s12962-024-00573-w (PMC11378520; doi:10.1186/s12962-024-00573-w)
Supplement: Supplementary file 3 — Supplementary Material 3: Supplemental Fig. 3 | LungRADs Score Descriptions: This file describes each LungRADs score (0, 1, 2, 3, 4a, 4b, 4x, Su and S) with corresponding clinical findings and case management. [file 12962_2024_573_MOESM3_ESM.pdf]

American College of Radiology™

Lung-RADS® v2022

Release Date: November 2022

Lung-RADS	Category Descriptor	Findings	Management
**0**	**Incomplete**<br>Estimated Population Prevalence: ~ 1%	Prior chest CT examination being located for comparison (see note 9)	Comparison to prior chest CT;
		Part or all oflungs cannot be evaluated	Additional lung cancer screening CT imaging needed;
		Findings suggestive of an inflammatory or infectious process (see note 10)	1-3 month LDCT
**1**	**Negative**<br>Estimated Population Prevalence: 39%	**No lung nodules OR**	12-month screening LDCT
		**Nodule with benign features:**<br>• Complete, central, popcorn, or concentric ring calcifications **OR**<br>• Fat-containing	
**2**	**Benign -** Based on imaging features or indolent behavior<br>Estimated Population Prevalence: 45%	**Juxtapleural nodule:**<br>• < 10 mm (524 mm^3) mean diameter at baseline or new **AND**<br>• Solid; smooth margins; and oval, lentiform, or triangular shape	
		**Solid nodule:**<br>• < 6 mm (< 113 mm^3) at baseline **OR**<br>• New < 4 mm (< 34 mm^3)	
		**Part solid nodule:**<br>• < 6 mm total mean diameter (< 113 mm^3) at baseline	
		**Non solid nodule (GGN):**<br>• < 30 mm (< 14,137 mm^3) at baseline, new, or growing **OR**<br>• ≥ 30 mm (≥ 14,137 mm^3) stable or slowly growing (see note 7)	
		**Airway nodule,** subsegmental - at baseline, new, or stable (see note 11)	
		Category 3 lesion that is stable or decreased in size at 6-month follow-up CT **OR** Category 4B lesion proven to be benign in etiology following appropriate diagnostic workup	
**3**	**Probably Benign -** Based on imaging features or behavior<br>Estimated Population Prevalence: 9%	**Solid nodule:**<br>• ≥ 6 to < 8 mm (≥ 113 to < 268 mm^3) at baseline **OR**<br>• New 4 mm to < 6 mm (34 to < 113 mm^3)	6-month LDCT
		**Part solid nodule:**<br>• ≥ 6 mm total mean diameter (≥ 113 mm^3) with solid component < 6 mm (< 113 mm^3) at baseline **OR**<br>• New < 6 mm total mean diameter (< 113 mm^3)	
		**Non solid nodule (GGN):**<br>• ≥ 30 mm (≥ 14,137 mm^3) at baseline or new	
		**Atypical pulmonary cyst:** (see note 12)<br>• Growing cystic component (mean diameter) of a thick-walled cyst	
		Category 4A lesion that is stable or decreased in size at 3-month follow-up CT (excluding airway nodules)	
**4A**	**Suspicious**<br>Estimated Population Prevalence: 4%	**Solid nodule:**<br>• ≥ 8 to < 15 mm (≥ 268 to < 1,767 mm^3) at baseline **OR**<br>• Growing < 8 mm (< 268 mm^3) **OR**<br>• New 6 to < 8 mm (113 to < 268 mm^3)	3-month LDCT;<br>PET/CT may be considered if there is a ≥ 8 mm (≥ 268 mm^3) solid nodule or solid component
		**Part solid nodule:**<br>• ≥ 6 mm total mean diameter (≥ 113 mm^3) with solid component ≥ 6 mm to < 8 mm (≥ 113 to < 268 mm^3) at baseline **OR**<br>• New or growing < 4 mm (< 34 mm^3) solid component	
		**Airway nodule,** segmental or more proximal - at baseline (see note 11)	
		**Atypical pulmonary cyst:** (see note 12)<br>• Thick-walled cyst **OR**<br>• Multilocular cyst at baseline **OR**<br>• Thin- or thick-walled cyst that becomes multilocular	
**4B**	**Very Suspicious**<br>Estimated Population Prevalence: 2%	**Airway nodule,** segmental or more proximal - stable or growing (see note 11)	Referral for further clinical evaluation
		**Solid nodule:**<br>• ≥ 15 mm (≥ 1767 mm^3) at baseline **OR**<br>• New or growing ≥ 8 mm (≥ 268 mm^3)	Diagnostic chest CT with or without contrast;<br>PET/CT may be considered if there is a ≥ 8 mm (≥ 268 mm^3) solid nodule or solid component;<br>tissue sampling;<br>and/or referral for further clinical evaluation<br>Management depends on clinical evaluation, patient preference, and the probability of malignancy (see note 13)
		**Part solid nodule:**<br>• Solid component ≥ 8 mm (≥ 268 mm^3) at baseline **OR**<br>• New or growing ≥ 4 mm (≥ 34 mm^3) solid component	
		**Atypical pulmonary cyst:** (see note 12)<br>• Thick-walled cyst with growing wall thickness/nodularity **OR**<br>• Growing multilocular cyst (mean diameter) **OR**<br>• Multilocular cyst with increased loculation or new/increased opacity (nodular, ground glass, or consolidation)	
		**Slow growing solid or part solid nodule** that demonstrates growth over multiple screening exams (see note 8)	
**4X**	Estimated Population Prevalence: < 1%	Category 3 or 4 nodules with additional features or imaging findings that increase suspicion for lung cancer (see note 14)	
**S**	**Significant or Potentially Significant**<br>Estimated Population Prevalence: 10%	**Modifier:** May add to category 0-4 for clinically significant or potentially clinically significant findings unrelated to lung cancer (see note 15)	As appropriate to the specific finding

© 2022 American College of Radiology® | All rights reserved

NOTES

1. **Lung-RADS Category:** Each exam should be coded 0-4 based on the nodule with the highest degree of suspicion.
2. **Lung-RADS Management:** The timing of follow-up imaging is from the date of the exam being interpreted. For example, 12-month screening LDCT for Lung-RADS 2 is from the date of the current exam. Also note that management of 4A lesions follows a stepped approach based upon follow-up stability or decrease in size.
3. **Practice Audit Definitions:** A negative screen is defined as categories 1 and 2; a positive screen is defined as categories 3 and 4. A negative screen does not mean that an individual does not have lung cancer.
4. **Nodule Measurement:** To calculate nodule mean diameter, measure both the long and short axis to one decimal point in mm, and report mean nodule diameter to one decimal point. The long and short axis measurements may be in any plane to reflect the true size of the nodule. Volumes, if obtained, should be reported to the nearest whole number in mm^3.
5. **Size Thresholds:** Apply to nodules at first detection and that enlarge, reaching a higher size category. When a nodule crosses a new size threshold for other Lung-RADS categories, even if not meeting the definition of growth, the nodule should be reclassified based on size and managed accordingly.
6. **Growth:** An increase in mean diameter size of > 1.5 mm (> 2 mm^3) within a 12-month interval.
7. **Slow Growing Non Solid (Ground Glass) Nodules:** A ground glass nodule (GGN) that demonstrates growth over multiple screening exams but does not meet the > 1.5 mm threshold increase in size for any 12-month interval may be classified as a Lung-RADS 2 until the nodule meets findings criteria of another category, such as developing a solid component (then manage per part solid nodule criteria).
8. **Slow Growing Solid or Part Solid Nodules:** A solid or part-solid nodule that demonstrates growth over multiple screening exams but does not meet the > 1.5 mm threshold increase in size for any 12-month interval is suspicious and may be classified as a Lung-RADS 4B. Slow growing nodules may not have increased metabolic activity on PET/CT; therefore, biopsy, if feasible, or surgical evaluation may be the most appropriate management recommendation.
9. **Prior Exams:** If waiting on prior exams (either a prior screening or diagnostic CT), the Lung-RADS 0 category is temporary until the comparison study is available and a new Lung-RADS category is assigned.
10. **Suspected Infectious or Inflammatory Findings:**
    a. Lung-RADS 0 with 1-3 month follow-up LDCT may be recommended for pulmonary findings suggesting an indeterminate infectious or inflammatory process. Such findings may include segmental or lobar consolidation, multiple new nodules (more than six), large solid nodules (≥ 8 mm) appearing in a short interval, and new nodules in certain clinical contexts (e.g. immunocompromised patient). At 1-3 month follow-up, a new Lung-RADS classification and management recommendation should be provided based on the most suspicious nodule.
    b. New solid or part solid nodules with imaging features more concerning for malignancy than an infectious or inflammatory process meeting Lung-RADS 4B size criteria may be classified as such with appropriate diagnostic and/or clinical evaluation.
    c. Some findings indicative of an infectious or infectious process may not warrant short-term follow-up (e.g. tree-in-bud nodules or new < 3 cm ground glass nodules). These nodules may be evaluated using existing size criteria with a Lung-RADS classification and management recommendation based on the most suspicious finding.
11. **Airway Nodules:**
    a. Endotracheal or endobronchial abnormalities that are segmental or more proximal are classified as Lung-RADS 4A.
    b. Subsegmental and/or multiple tubular endobronchial abnormalities favor an infectious process; if no underlying obstructive nodule is identified, these lesions may be classified as Lung-RADS 0 (likely infectious or inflammatory) or 2 (benign).
    c. The presence of air in segmental or more proximal airway abnormalities often favors secretions; if no underlying soft tissue nodule is identified, these findings may be classified as Lung-RADS 2.
    d. Segmental or more proximal airway nodules that persist on 3-month follow-up CT are upgraded to Lung-RADS 4B with management recommendation for further clinical evaluation (typically bronchoscopy).
12. **Atypical Pulmonary Cysts:**
    a. Thin-walled Cyst: Unilocular with uniform wall thickness < 2 mm. Thin-walled cysts are considered benign and are not classified or managed in Lung-RADS.
    b. Thick-walled Cyst: Unilocular with uniform wall thickness, asymmetric wall thickening, or nodular wall thickening ≥ 2 mm (cystic component is the dominant feature); manage as an atypical pulmonary cyst.
    c. Multilocular Cyst: Thick or thin-walled cyst with internal septations. Manage as an atypical pulmonary cyst.
    d. Cavitary Nodule: Wall thickening is the dominant feature; manage as a solid nodule (total mean diameter).
    e. Cyst with an Associated Nodule: Any cyst with adjacent internal (endophytic) or external (exophytic) nodule (solid, part-solid, or ground glass). Management is based upon Lung-RADS criteria for the most concerning feature.
    f. Growth: > 1.5 mm increase in nodule size (mean diameter), wall thickness, and/or size of the cystic component (mean diameter) occurring within a 12-month interval.
    g. Fluid-containing cysts may represent an infectious process and are not classified in Lung-RADS unless other concerning features are identified.
    h. Multiple cysts may indicate an alternative diagnosis such as Langerhans cell histiocytosis (LCH) or lymphangioleiomyomatosis (LAM) and are not classified in Lung-RADS unless other concerning features are identified. (Reference: Seaman DM, Meyer CA, Gilman MD, McCormack FX. Diffuse Cystic Lung Disease at High-Resolution CT. AJR 2011;196: 1305-1311)
13. **Category 4B:** Management is predicated on clinical evaluation (comorbidities), patient preference, and risk of malignancy. Radiologists are encouraged to use the McWilliams, et al Assessment Tool when making recommendations (https://brocku.ca/lung-cancer-screening-and-risk-prediction/risk-calculators/).
14. **Category 4X:** Category 3 or 4 nodules with additional imaging findings that increase the suspicion of lung cancer, such as spiculation, lymphadenopathy, frank metastatic disease, a GGN that doubles in size in 1 year, etc. 4X is a distinct Lung-RADS category; X should not be used as a modifier.
15. **Exam Modifier:** An S modifier may be added to Lung-RADS categories 0-4 for clinically significant or potentially clinically significant findings unrelated to lung cancer.
    a. Management should adhere to available ACR Incidental Findings management recommendations (https://www.acr.org/Clinical-Resources/Incidental-Findings). The ACR Lung Cancer Screening CT Incidental Findings Quick Reference Guide summarizes common findings and management (https://www.acr.org/-/media/ACR/Files/Lung-Cancer-Screening-Resources/LCS-Incidental-Findings-Quick-Guide.pdf).
    b. Findings that are already known, and have been or are in the process of clinical evaluation DO NOT require an S-modifier. Any evidence of a concerning change in a known significant or potentially significant finding that is unexpected warrants renewed use of the S-modifier.
16. **Lung Cancer Diagnosis:** Once a patient is diagnosed with lung cancer, further management (including additional imaging such as PET/CT) may be performed for purposes of lung cancer staging; this is no longer considered screening.

Abbreviations: LDCT: low dose chest CT; GGN: ground glass nodule

Additional resources available at: https://www.acr.org/Clinical-Resources/Reporting-and-Data-Systems/Lung-Rads

© 2022 American College of Radiology® | All rights reserved
